# Progress in Advanced Ceramic Fibers: From Spinning Techniques to Frontier Applications

**DOI:** 10.3390/ma19173573

**Published:** 2026-08-23

**Authors:** Huihui Yan, Chun Xiang, Heng Qian, Chaoqian Zhao

**Affiliations:** 1 School of Mechanical Engineering, Zhejiang University of Water Resources and Electric Power, Hangzhou 310018, China; xiangchun@zuwe.edu.cn (C.X.); qianh@zuwe.edu.cn (H.Q.); 2 Key Laboratory of Key Technologies for Mechanical Industry Hydroelectric Power Generation Pump Turbine, Hangzhou 310018, China; 3 Institute of Laser Advanced Manufacturing, Zhejiang University of Technology, Hangzhou 310014, China

**Keywords:** ceramic fibers, structural and functional design, spinning process, frontier applications

## Abstract

Although ceramic materials exhibit excellent thermal stability, high melting points, and chemical inertness, their intrinsic brittleness restricts their application across various fields. To address this challenge, ceramic fibers possessing the flexibility and functionality demanded by advanced applications have emerged. This review provides an overview of recent progress in ceramic fibers, emphasizing four major spinning techniques, including melt spinning, electrospinning, solution blow spinning, and wet spinning, along with their underlying fabrication mechanisms and process–structure relationships. The fibrous architectures (including aerogels, textiles, and membranes) demonstrate exceptional performance in thermal protection, extreme environment, wave absorption, thermoelectric energy conversion, and wearable electronic textiles and high-temperature catalysis. Despite these advancements, challenges remain in scalable continuous production, long-term stability under realistic service conditions, multifunctional integration, and cost-effective sustainability. This review provides a roadmap for translating laboratory innovations into practical, large-scale deployment in aerospace, energy, and electronic systems.

## 1. Introduction

Ceramic materials, renowned for their exceptional chemical inertness, high melting points, and superior thermal stability, have long served as indispensable foundational components in a wide range of fields, including aerospace, energy, chemical engineering, construction, and advanced electronics [1,2,3,4,5,6,7]. Even so, the intrinsic brittleness of conventional bulk ceramics results in inadequate fracture toughness and poor mechanical strength [8,9,10]. These fundamental limitations often lead to catastrophic failure under impact or complex mechanical deformation, which severely constrains their applications in dynamic or flexible environments [1,11,12]. In response to these challenges, research has increasingly focused on one-dimensional ceramic fibers [9,13,14]. By reducing the dimensionality to the micro- or nanoscale, these fibers not only retain the thermal stability of their bulk counterparts but also exhibit remarkable structural flexibility [1,2,3,8]. This transition is primarily attributed to the refined grain structure and diminished cross-sectional area, which effectively suppress crack propagation pathways [1,15,16]. As a result, the materials acquire unexpected, polymer-like characteristics, including flexibility, stretchability, and elasticity, enabling their integration into textiles and composite systems where traditional ceramics would be unsuitable [1,11,17].

The evolution of ceramic fiber materials has been driven by the need for ever-higher thermal/chemical stability and functional versatility, pushing their compositional landscape from conventional oxides (Al_2_O_3_, ZrO_2_, mullite) through non-oxide systems (e.g., SiC, TiC, ZrB_2_) to emerging high-entropy ceramic (HEC) formulations [3,17,18,19,20,21,22,23]. Recent breakthroughs have propelled ceramic fibers beyond their traditional role as merely passive reinforcements in composites, undergoing a paradigm shift, progressively evolving into intelligent and multifunctional platforms [24,25,26,27,28]. Enabled by responsive mechanisms such as sensing, actuation, and adaptive thermal management, ceramic fibers are now poised for integration into next-generation flexible electronics, soft robotics, and extreme-environment smart textiles [1,29,30]. This compositional, functional and application diversification inevitably raises the bar for processing: as phase purity, grain growth, and defect tolerance become decisive, macroscopic properties and usable application windows are no longer inherent to the chemistry alone but are predominantly dictated by microstructure—which, in turn, is set during fabrication [30,31]. Direct physical deposition involves the natural formation of 1D morphologies via the growth of atoms/molecules along preferred orientations, including Physical Vapor Deposition (PVD), Molecular Beam Epitaxy, and Pulsed Laser Deposition—while direct chemical deposition relies on chemical reactions, such as Chemical Vapor Deposition (CVD), hydrothermal synthesis, and sol–gel processes [1,7,9,15,27]. While these methods offer high precision, spinning techniques are recognized as the most productive approach for industrial-scale manufacturing [9,32,33,34,35].

In recent years, significant innovations in microfabrication technology have fundamentally transformed the manufacturing landscape of ceramic fibers [35,36,37]. Central to this transformation is the shift from conventional melt-spinning methods towards more refined, precise, and controllable routes, such as electrospinning, solution blow spinning, and wet spinning [18,29,35,38]. These technological evolutions have enabled an unprecedented level of fabrication control over microstructure, size, quality and efficiency of ceramic fibers. In parallel, taking inspiration from natural architectures, researchers have gained precise mastery over both the microscopic morphology and macroscopic assembly of these fibers [6,39,40]. This has opened up novel and unprecedented property spaces by integrating sophisticated biomimetic designs, such as a hierarchical structure displayed in spider silk, brick-and-mortar microstructure observed in pearl shell, or the helical buckling of ivy, with advanced physics concepts like high-entropy engineering and double-negative metamaterials [11,15,16,23,41,42]. Consequently, this review focuses specifically on the evolution and mechanisms of these spinning techniques.

**Figure 1:**
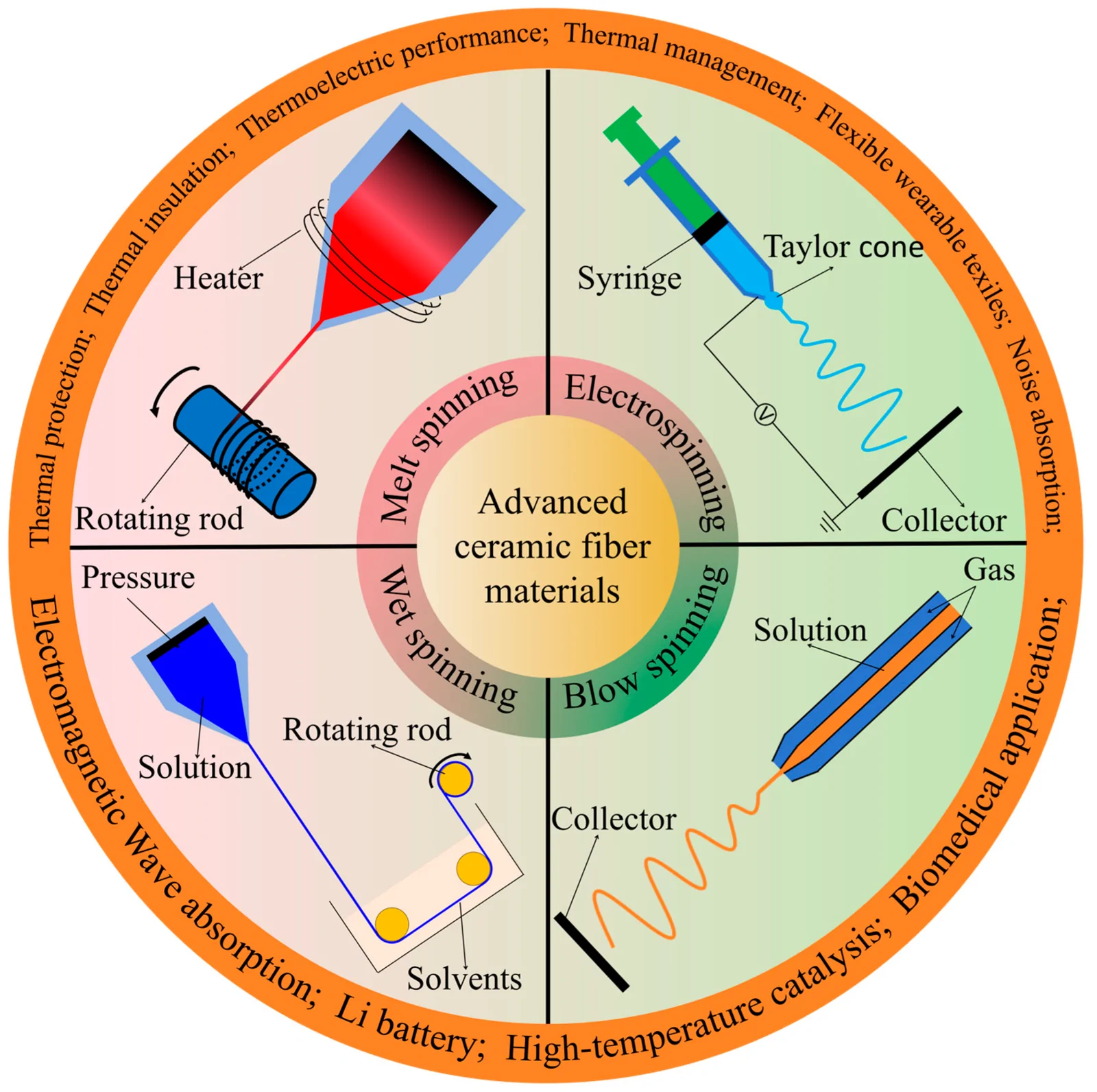
Spinning technologies and applications of advanced ceramic fibers.

In selecting studies, focus was placed on publications pertinent to spinning technologies. Priority was given to research articles published within the last five years to ensure timeliness. High-impact studies were deliberately selected to reflect the field’s cutting edge, drawing from leading journals such as *Science*, *Nature* and their sister journals, *Advanced Materials*, *Advanced Functional Materials*, *Materials Today*, *Chemical Reviews*, *Progress in Materials Science*, etc. Furthermore, broad database coverage was ensured, including major platforms such as Elsevier, Wiley, Springer Nature, ACS, etc. In light of these rapid advancements, this review aims to provide a comprehensive and critical discussion structured around three key dimensions: (i) the fabrication methodologies governing fiber formation, (ii) the strategic regulation of microstructure to tailor mechanical and functional properties, and (iii) the exploration of cutting-edge application scenarios (Figure 1). By synthesizing these topics, the primary objective of this review is to provide readers with a comprehensive overview of the latest research landscape in ceramic fibers and to offer forward-looking insights that may inspire innovative pathways for the future development of this essential class of materials.

## 2. Fabrication Techniques of Ceramic Fibers

### 2.1. Electrospinning

Electrospinning uses a high-voltage electric field to overcome surface tension, generating a Taylor cone and stretching the jet into nanofibers [1,15,35,43]. While this technique offers unparalleled control over fiber diameter (down to tens of nanometers) and morphology, it suffers from low throughput (0.1–1.0 g/h) and the inability to produce continuous monofilaments—a fundamental limitation rooted in its point-to-point deposition mechanism [35]. In practice, ceramic fibers are typically derived from polymer solutions loaded with ceramic precursors or particles, allowing for the subsequent removal of the organic template via thermal treatment [27]. Post-fabrication, these nanofibers are often dispersed in an aqueous medium, cast into molds, and freeze-dried to construct ceramic aerogels [7]. Among the various fabrication strategies for fibers, electrospinning stands out due to its high throughput, versatility in feedstock selection, tunable fiber architectures, and compatibility with other manufacturing technologies [9]. Detailed electrospinning properties of ceramic fibers and their applications are shown in Table 1.

To overcome the intrinsic defects inherited from polymer templates and to tailor fiber architecture for extreme environments, foundational design strategies have evolved from compositional mixing to multiscale engineering. Foundational design strategies for advanced ceramic fibers have evolved from mere compositional mixing to sophisticated multiscale architecture engineering. At the molecular level, precursor engineering is pivotal for achieving multiphase synergy. For instance, the molecular compounding of carbon precursors with mullite (Al_2_O_3_-SiO_2_) sols yields hybrid carbon–ceramic nanofibers, where the carbon phase kinetically inhibits ceramic grain coarsening, while the ceramic matrix conversely stabilizes the carbon against oxidation [2]. Extending this concept, the high-entropy ((Gd_1/2_Lu_1/2_)_2_(Ti_1/3_Zr_1/3_Hf_1/3_)_2_O_7_) design principle utilizes equimolar multi-principal elements, often derived from molecularly homogeneous acetylacetonate complexes as precursors, to maximize configurational entropy, thereby constructing elastic oxide networks capable of withstanding hypersonic thermal shocks [23]. Beyond chemical composition, rheological and structural modulation dictates processability and final performance. Synthesizing aluminosilicate dopes via Al/Si hydrolysis, coupled with PVP-mediated viscosity control, ensures spinnability and results in flexible networks serving as fire-retardant skeletons [45]. More strikingly, tailored spinning fields and asymmetric contraction have enabled unique fiber morphologies. Turbulent-field electrospinning produces 3D entangled hypocrystalline zircon nanofibers with near-zero Poisson’s ratios [47], whereas eccentric electrospinning fibers (mullite, HfO_2_, ZrO_2_ and TiO_2_) mimics ivy tendril morphology through asymmetric solvent evaporation, optimizing thermal insulation [39]. Furthermore, aligning SiC fibers via high-speed orientation and integrating them into anisotropic aerogels demonstrates a pathway toward directional heat transfer management [48]. To address intrinsic material defects, self-templated electrospinning has emerged as a solution to circumvent traditional polymer template limitations. By modulating phase transitions, this strategy effectively suppresses high-temperature Al_2_O_3_ grain growth, yielding robust oxides for extreme environments [49]. Synchronously, the functionalization frontier has expanded beyond thermal protection. Recent advances demonstrate the fabrication of flexible Al_2_O_3_ membranes capable of passive daytime radiative cooling, effectively coupling solar reflectance with infrared emissivity to the atmospheric window [43].

Performance optimization for extreme environments is increasingly achieved through a multiscale design paradigm, evolving from atomic-level doping to hierarchical architecture construction. At the microstructural level, dopants and secondary phases are engineered to stabilize grain boundaries and tailor electrical properties. For instance, titanium incorporation into polycarbosilane precursors induces in situ TiC formation during pyrolysis at 1800 °C, effectively pinning SiC grain boundaries and enabling deep deoxidation to maintain structural integrity under extreme stress [18]. Similarly, ligand-regulated synthesis of linear TiO_2_ sols eliminates template-induced defects, where implanted nanoclusters trigger interfacial nucleation to ensure a uniform dispersion within crystalline/amorphous dual-phase matrices [30]. This precise control extends to electrical tunability; by modulating the carbothermal reduction degree of Si-C-N fibers at 1400 °C, researchers have achieved predictable resistivity gradients for optimized thermal protection [44]. Beyond bulk composition, heterophase interface design plays a critical role in high-temperature resilience. Encapsulating SiC nanoparticles within Al_2_O_3_–mullite self-templated fibers creates heterointerfaces that suppress grain coarsening, while surface oxide shells act as diffusion barriers against oxygen permeation, preserving single-fiber toughness up to 1500 °C [50]. Complementary to this, high-entropy engineering leverages one-dimensional induction molding to produce chromate nanofibers with amorphous domains, which, when paired with SiO_2_ matrices, exhibit enhanced flexibility for spacecraft thermal management [22]. Finally, hierarchical and functional integration addresses complex application demands. Sea–island nanostructures, combining carbon matrices with ceramic inclusions (C/SiCON), optimize electron and phonon transport for energy management [53]. Concurrently, wrapping flexible SiO_2_ nanofibers with reduced graphene oxide (rGO) yields entangled networks that synergize lightweight thermal resistance with broadband sound absorption [54]. At the atomic level, nitrogen doping facilitates the transformation of Fe nanoparticles into single-atom anchors within SiOC fibers, significantly boosting electromagnetic wave absorption [55]. Furthermore, sol–gel electrospinning coupled with room-temperature domino cascade reduction introduces defect states that impart fiber (TiO_2_, SnO_2_ and BaTiO_3_) conductivity, bridging the gap between ceramic processing and lithium battery applications [56].

The culmination of ceramic fiber development lies in multiscale assembly and multifunctional integration, transitioning from single-component optimization to system-level engineering. At the microscale, structural design focuses on stress relief and morphological control; for instance, coaxial electrospinning coupled with low-surface-energy solvent exchange mitigates capillary drying stresses, yielding flexible hollow SiO_2_ nanofibers essential for lightweight composites [51]. Building on this, compositional and stacking strategies are employed to tailor macroscale properties. By tuning the configurational entropy of SiO_2_-ZrO_2_ fibers and organizing them into ultra-thin multilayer stacks, synergistic optimization across multiple physical domains is achieved [52]. To bridge the gap between nanoscale materials and macroscopic applications, pre-tensioned knitted topological networks have emerged as load-bearing scaffolds. These are integrated with crosslinked SiO_2_ nanofiber networks via ice-templated freezing and calcination [21]. This hierarchical configuration leverages topological constraints to introduce multi-stable and pre-stress mechanisms, enabling exceptional deformation adaptability and energy dissipation for integrated thermal-impact protection systems. Finally, biomimetic assembly strategies extend these capabilities to wearable electronics. Combining ball milling and twisting with conventional electrospinning constructs orderly “brick-and-mortar”-structured oxide ceramics (TiO_2_, ZrO_2_ and SiO_2_), offering a robust platform for flexible energy devices and electronic textiles [11].

To bridge the gap between scientific exploration and commercial viability, future research should prioritize needleless electrospinning, high-speed roll-to-roll collection systems, or hybrid approaches that incorporate melt-derived continuous precursors prior to ceramic conversion.

### 2.2. Melt Spinning

Melt spinning is a mechanically driven process wherein raw materials are thermally melted and extruded through spinneret orifices under pressure. The resulting melt jets are subsequently attenuated and solidified via aerodynamic forces, gravity, and convective cooling to yield continuous filaments [27,57,58,59]. With reported production rates reaching approximately 50 g/h for dual-nozzle setups, roughly two orders of magnitude higher than laboratory-scale electrospinning, centrifugal spinning offers improved dimensional homogeneity and mechanical performance through controlled device geometry [15]. Fibers produced via this route typically exhibit relatively large diameters (generally ≥ 2 µm) and suffer from poor morphological controllability, alongside limitations regarding precursor selection [9]. Consequently, the driving force of melt spinning—mechanical extrusion—renders it highly efficient for macroscale production, yet inherently unstable and incapable of precise diameter control at submicron scales [1]. To address these challenges, many ceramic fibers are fabricated using a precursor-polymer pathway. In this strategy, a polymeric precursor (e.g., polycarbosilane) is melt-spun into “green” filaments; the desired ceramic phase is then obtained through subsequent curing, pyrolysis, and calcination. Detailed melt-spinning properties of ceramic fibers and their applications are shown in Table 2.

To mitigate shrinkage-induced defects and achieve flexibility despite brittleness, the design of high-performance inorganic fibers is fundamentally predicated on reconciling intrinsic brittleness with mechanical flexibility. A prevalent strategy involves exploiting unique microstructural features to activate plastic deformation mechanisms. For example, selecting “tough inorganic semiconductors” such as Ag_2_Te_0.6_S_0.4_, which feature coexisting amorphous and nanocrystalline domains, enables shear band deformation, thereby circumventing the brittle fracture limits inherent to conventional thermoelectric materials [60]. Similarly, in carbon-based systems, highly thermally conductive graphene fibers (GFs) serve as cores, leveraging their natural micro-wrinkles and oriented crystallites. This facilitates the construction of robust core–shell architectures with conformal ceramic shells (e.g., TiC), wherein cross-scale, fractal-like interlocking interfaces decouple thermal conduction from oxidation resistance [29]. This materials-first paradigm shifts the failure mode from catastrophic cracking to energy-dissipative deformation.

To translate these material designs into scalable, continuous fibers, advanced processing routes are employed to dynamically engineer interfaces and phases. Techniques such as the molten core method and the glass-cladding template combined with two-step thermal drawing utilize fluid-state co-drawing and controlled thermal stress to induce functional textures, for example, (00L)-plane-oriented Bi_2_Te_3_ nanosheets for enhanced carrier transport, while preserving structural integrity [61]. Furthermore, for functional glasses, a decoupled approach involving melt drawing of amorphous precursors followed by in situ-controlled crystallization is adopted. Guided by phase diagrams and molecular dynamics (MD) simulations, this method enables the precipitation of selective nanocrystals (e.g., Ba_2_GdF_7_:Tb^3+^) within the fiber core, conferring scintillation activity upon the fiber without compromising its waveguiding transparency [62]. Collectively, these principles demonstrate that the combination of tailored microstructures with precise thermal–mechanical processing facilitates the scalable fabrication of inorganic fibers that simultaneously achieve ultra-flexibility and high functionality.

Melt spinning inherently encodes the genetic blueprint for continuous industrial-scale production; however, its full potential is currently constrained by precursor limitations and significant shrinkage defects during the ceramization process. The core challenge lies not in the spinnability of the polymer itself, but in controlling dimensional stability and defect inheritance throughout the curing and pyrolysis stages. Addressing volumetric shrinkage—which often exceeds 30%—and suppressing the propagation of microcracks inherited from the polymer precursor are essential prerequisites for the fabrication of high-performance ceramic fibers via this route.

### 2.3. Blow Spinning

Blow spinning is an economical and highly efficient fiber fabrication technology capable of producing a broad spectrum of materials, including ceramic, metal, and polymer fibers [1,15,69,70,71]. Positioned as a high-throughput alternative to electrospinning, blow spinning utilizes aerodynamic forces to achieve rapid fiber formation. This technique can produce nanofibers at a throughput of 6 kg/h by multiple jets, providing the capability for mass production [35,63]. Compared to conventional electrospinning, blow spinning offers significantly higher productivity, rendering it particularly attractive for large-scale manufacturing of continuous ceramic nanofibers and their three-dimensional assemblies [7]. This combination of scalability, versatility, and cost-effectiveness positions blow spinning as a promising alternative for advanced ceramic aerogel production. Detailed blow-spinning fabrication of ceramic fibers and their applications are shown in Table 2.

The fundamental strategy for reconciling the intrinsic brittleness of ceramics with structural flexibility lies in geometric downscaling and hierarchical microstructure engineering [3,17]. By reducing the building blocks to the nanoscale, the dominant failure mode shifts from catastrophic crack propagation to energy-dissipative mechanisms governed by fiber buckling, interfacial sliding, and elastic recoil. This paradigm is actualized through distinct yet complementary architectural innovations. First, bio-inspired heterogeneous cores mimic the dense protective sheath and porous medullary layer of animal hair, as exemplified by Al_2_O_3_/SiO_2_ core–shell structures that balance surface hardness with internal compliance [6]. Second, intrinsic ductility is engineered at the microstructural level via nanograin–glass dual-phasic mullite fibers, where a compliant glassy matrix accommodates plastic strain while embedded nanograins arrest shear localization [19]. Third, sacrificial templating utilizes polymeric scaffolds (e.g., PVB, PVA) to create hybrid networks that are subsequently pyrolyzed into functional metal oxides or metals (In-Ga-Zn oxide and CuO), locking in porosity and flexibility [65]. Collectively, these multiscale strategies synergistically weave mechanical robustness with advanced functionalities, transforming fragile filaments into resilient, three-dimensionally entangled networks.

To translate these designs into macroscopic materials, advanced blow-spinning techniques replace electrostatic fields with purely aerodynamic forces [3,17]. By employing needleless roll-to-roll systems or high-speed vertical airflows, strong gas shear stresses and periodic Kármán vortex streets are generated, which drive Taylor cone formation and jet instability, thereby enabling stable, high-throughput production. Specific chemical systems facilitate this process: PVA-TEOS-AlCl_3_ sol–gel chemistry accelerates gelation and yields SiO_2_-Al_2_O_3_ composite phases, leading to anisotropic, layered microfiber networks with enhanced moduli [64]. Similarly, PVA-mediated solutions containing AlCl_3_·6H_2_O enable the continuous deposition of wet fiber skeletons that, after controlled pyrolysis, lock Al_2_O_3_ in a non-crystallized or weakly crystallized nano-grained state (γ-Al_2_O_3_), retaining flexibility and high specific surface area [66,67]. These processes collectively ensure the structural integrity of the fibers throughout the entire transformation from precursor to final ceramic form.

However, the inherently chaotic nature of the turbulent gas field results in a broad fiber diameter distribution and frequent defects, compromising the mechanical isotropy of the final assembly. A critical gap in the existing literature is the absence of standardized statistical characterization protocols for diameter distribution and structural homogeneity. Establishing quantitative correlations between gas flow dynamics and fiber morphology is therefore essential for effective quality control.

### 2.4. Wet Spinning

Wet spinning is a versatile technique for fabricating continuous functional fibers [20,33,72,73,74]. In this process, a spinning solution is extruded through a spinneret into a coagulation bath, followed by solvent exchange, chemical or ionic crosslinking, and post-treatment, ultimately yielding self-supporting, machine-woven fibers with customizable structures [74]. Wet spinning is particularly well suited for producing continuous (4–5 g/h) [75], weaveable fibers with sophisticated core–sheath architectures. Its irreplaceable advantage lies in simultaneously achieving high functional integration and compatibility with textile processing. This adaptability renders wet spinning a powerful platform for integrating diverse functional components into continuous macroscopic fibers. Detailed wet-spinning fabrication of ceramic fibers and their applications are shown in Table 2.

To overcome the brittleness of ceramics while maintaining spinnability, nanoscale building blocks are tailored to emulate polymer-like flexibility. The design of flexible inorganic fibers often begins with tailoring the building blocks at the nanoscale to emulate the flexibility of polymeric chains. By utilizing sub-1 nm, high-aspect-ratio GdOOH nanowires as orientable assembly units, wet spinning enables precise control over the flow field—integrating injection stress, shear, solvent exchange, and gravity—to form a spring-like superlattice structure. This architecture converts tensile strain into the progressive straightening and limited sliding of nanowires rather than brittle fracture, achieving remarkable stretchability and elasticity [16]. Extending this principle to the microscale, coaxial wet spinning integrates functional ceramic cores with protective shells. For instance, surface-modified Al-doped ZnO nanoparticles serve as an identifiable conductive core, encapsulated by a sheath of aramid nanofibers. Subsequent cold isostatic pressing consolidates the structure, wherein the synergy of shell confinement, dense particle contact networks, and interfacial shear lag dissipation transforms intrinsically brittle ceramics into high-strength, large-strain, and knittable electronic-skin (e-skin) fibers [68]. For applications demanding extreme thermal and mechanical stability, fibrous monolithic architectures provide a solution through engineered interfaces. Specifically, ZrB_2_-SiC ultra-high-temperature ceramics are formulated into a polymer-bound, spinnable slurry serving as the “cell”, while a weak interface layer (comprising SiC whiskers or BN) is concurrently prepared as a concentric slurry [20]. Through co-extrusion wet spinning, core–sheath fiber precursors are continuously produced, ensuring uniform interface adhesion and eliminating strength gradients that would otherwise arise from repetitive infiltration. Following debinding and hot pressing, the resulting structure exploits the weak interface to induce crack deflection and cell pull-out for toughening, while the high-thermal-conductivity SiC interface enhances heat dissipation and ablation resistance. Collectively, these strategies demonstrate that the integration of nanoscale assembly with macroscale structural design enables the transformation of intrinsically brittle ceramics into fibers capable of withstanding both extreme deformation and harsh environments.

Hence, the primary challenge lies not in the ability to form a filament per se, but rather in achieving stable extrusion combined with controlled, defect-free solidification kinetics within the coagulation bath.

## 3. Multifunctional and Frontier Applications

Owing to their unique structures and outstanding comprehensive properties, ceramic fibers and their macroscopic assemblies (e.g., aerogels, textiles, etc.) serve an indispensable role in numerous cutting-edge fields.

### 3.1. Thermal Protection and Superinsulation

Modern thermal protection in aerospace and extreme-environment engineering increasingly demands materials that can simultaneously suppress heat ingress, withstand severe thermomechanical and oxidative transients, and bear or conform to structural loads [2,20,68]. Conventional solutions remain constrained by a classic trade-off: rigid ultra-high-temperature ceramics resist ablation but fracture under strain or thermal shock, whereas lightweight porous insulators avoid cracking yet soften, coarsen, or oxidize above approximately 1200–1500 °C [2,20]. Recent progress redefines this challenge through multiscale architecture and multiphase synergy, rather than compositional optimization alone. Super-insulating nanofibrous aerogels demonstrate that the combination of nanoporosity, lamellar or cellular topology, and locally closed-pore or high-entropy-stabilized phases enables ultra-low thermal conductivity while maintaining reversible compressibility and oxidative stability up to approximately 1500–1600 °C [2,23]. For surface and civil/industrial substrates, dual-aerogel ceramic coatings show that pairing a nanoporous insulating phase with a nanofibrous “flexible skeleton” restores bending-fatigue resistance and adhesion, delivering fire superprotection at thicknesses of only a few millimeters [45]. Where heat flux is so intense that localized hot spots dominate, “dredging” designs employ highly conductive carbon/ceramic cores with robust ceramic shell interfaces (graphene fibers with TiC coating) to spread heat laterally and protect the core, thereby lowering peak surface temperature and mass loss under flames exceeding 2000 °C [29]. At the systems level, rigid Si-C-N foam-ceramic/metamaterial hybrids incorporate hierarchical pore engineering and periodic intercalation to unify thermal insulation, load-bearing capacity, and even extreme-temperature electromagnetic wave management [44]; meanwhile, fibrous monolithic ZrB_2_–SiC architectures illustrate how weak interfacial layers and brick-and-mortar topologies enhance crack tolerance and plasma-ablation stability [20]. Taken together, these advances suggest that next-generation thermal protection systems will hinge on engineered interfaces, pore-scale heat-transfer control, and multiphase stability windows, rather than on any single “best” ceramic composition.

**Figure 2:**
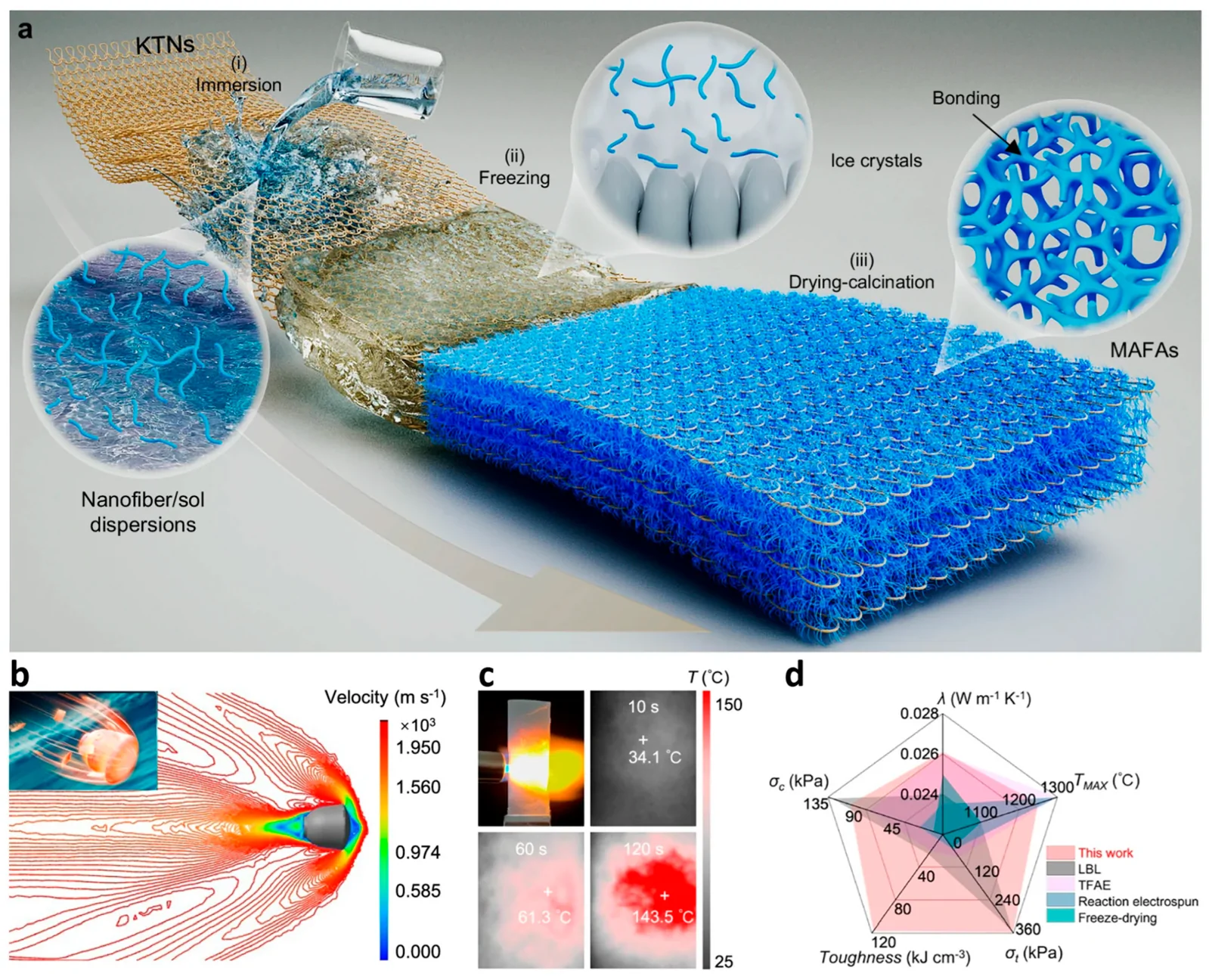
Ceramic mechanically adaptable fibrous aerogels for thermal insulation. (**a**) Schematic illustration of the fabrication process of mechanically adaptable fibrous aerogels. (**b**) Computational fluid dynamics simulation illustrating the velocity distribution nephogram of the re-entry capsule within a wind tunnel environment. (**c**) Optical and infrared images of the fibrous aerogels after being subjected to a butane blow torch for 120 s. (**d**) A radar plot comparing the fibrous aerogels and other aerogel-like materials (reprinted with permission from Ref. [21]).

Effective thermal management is pivotal to the reliability of high-power-density electronics and aerospace systems, yet conventional materials are often plagued by inherent trade-offs between thermal conductivity, mechanical robustness, and functional versatility [8,21,51,52,61]. Architectured ceramic nanofibers and aerogels have emerged as a disruptive solution to this trilemma. First, mechanical resilience has been revolutionized through microstructural control. Self-templated electrospinning yields dense, defect-free SiO_2_ fibers that surmount the intrinsic brittleness of ceramics, achieving exceptional tensile strength (1.41 GPa) and toughness (34.29 MJ m^−3^) [8]. This mechanical superiority extends to dynamic environments; knitted topological frameworks integrate SiO_2_ fibrous aerogels to withstand thermal shock fatigue for over 500 cycles without degradation [52]. Second, extreme-environment heat dissipation has been redefined by interfacial engineering. SiO_2_ aerogel-based vapor percolators utilize hierarchical nano/micro tunnels to suppress the Leidenfrost effect up to 1000 °C, facilitating an ultra-high heat flux of 110.43 W cm^−2^ (Figure 2) [21]. Parallel to heat removal, energy harvesting and stealth functionalities have been seamlessly integrated. Thermal drawing and interfacial engineering produce flexible thermoelectric Bi_2_Te_3_ fibers with a peak ZT of 1.4 at 300 K for waste-heat recovery [61]. Concurrently, high-entropy silicon–zirconium aerogels leverage configurational entropy to synergistically optimize stealth (RL = −26.6 dB), mechanics (335.5 kPa), and thermal insulation (λ = 0.034 W m^−1^ K^−1^) [51]. Finally, the decoupling of phonon–electron transport represents the frontier of thermal regulation. Carbon–ceramic nonwovens with sea–island nanostructures (C/SiCON) achieve an ultra-low cross-plane thermal conductivity (19.8 mW m^−1^ K^−1^) while maintaining high electrical conductivity (4.2 S cm^−1^) [53]. Complementing this, high-entropy chromate metafabrics ((La_0.2_Y_0.2_Nd_0.2_Gd_0.2_Sr_0.2_)CrO_3_) offer broadband infrared emissivity (97.1%) for radiative cooling in space applications [22]. Collectively, these innovations demonstrate a paradigm shift from monolithic materials to multifunctional, architectured systems.

Effective thermal insulation under extreme conditions has evolved beyond mere thermal resistance, now demanding architectures that concurrently exhibit ultra-low conductivity, high-temperature stability, and mechanical compliance [3,17,46,47,48]. While classical nanoporous aerogels approach the theoretical limit of conduction suppression, their susceptibility to brittle catastrophic failure limits practical deployment. To circumvent this, three-dimensional (3D) nanofibrous sponges replace weak point-to-point contacts with entangled high-aspect-ratio networks, tolerating large compressive strains and fatigue while maintaining conductivity within 0.027–0.033 W m^−1^ K^−1^ [3,17]. Addressing the entrenched mechanics–insulation trade-off, La_2_Y_0.4_TiZr_2_O_9.6_ core–sheath nanofibrous aerogels decouple functionality: a flexible nanofibrous core provides reversible deformability, while a nanoporous ceramic sheath enforces Knudsen-scale pore confinement. This configuration retains superinsulation below stationary air (<24 mW m^−1^ K^−1^) without sacrificing bending or compressive compliance [46]. Further pushing the deformation limits, bio-inspired highly buckled oxide ceramic (Mullite, HfO_2_, ZrO_2_ and TiO_2_) nanofibers convert lateral slack into axial elongation, enabling record tensile strains while rendering insulation nearly insensitive to stretching—a critical attribute for morphing skins or vibrating subsystems [39]. For the most demanding thermal shock regimes, hypocrystalline zircon nanofibrous aerogels architected into zig-zag layouts realize near-zero Poisson’s ratio and near-zero thermal expansion. These characteristics suppress thermomechanical damage, and, aided by residual carbon, maintain stability with thermal conductivity of ~104 mW m^−1^ K^−1^ at 1000 °C [47]. Beyond isotropic resistance, anisotropic SiC@SiO_2_ fiber aerogels align one-dimensional pathways to direct heat flux rather than merely block it, reporting transverse conductivity as low as ~0.018 W m^−1^ K^−1^ with anisotropy factors exceeding 5, while maintaining oxidative integrity up to 1300 °C [48]. Crucially, bridging lab-scale performance to industrial relevance, needle-free Kármán vortex blow spinning demonstrates kilogram-scale output without compromising ceramic (SiO_2_, Al_2_O_3_ ZrO_2_ and TiO_2_) fiber quality [63]. Collectively, these advances reframe thermal insulation as a multiscale co-optimization problem, where pore-scale transport physics, architectured deformability, and scalable synthesis must be harmonized rather than traded off.

Despite decades of progress, the simultaneous achievement of ultra-low thermal conductivity, mechanical compliance, and long-term oxidative stability above 1500 °C remains an elusive goal—a gap that recent multiscale architectures attempt to bridge but have yet to fully close. A primary obstacle lies in the disconnect between laboratory characterization and realistic service conditions. While ceramic fibers demonstrate exceptional thermal stability and ablation resistance under short-term static or quasi-static tests, the coupled effects of thermal shock, aerodynamic shear, and oxidative erosion under realistic hypersonic flight environments remain poorly understood. In particular, during re-entry or hypersonic cruise, the combined thermomechanical loading frequently induces interfacial debonding between fibers and the underlying substrate—a failure mechanism seldom captured by conventional testing protocols. A second fundamental challenge stems from the inherent trade-off between thermal conductivity and insulation. Highly crystalline fibers offer superior mechanical strength but exhibit higher thermal conductivity, whereas amorphous fibers provide better insulation yet are prone to devitrification and shrinkage at ultra-high temperatures. Finally, lifecycle costs associated with precursor synthesis (e.g., polycarbosilane purification) and multi-step pyrolysis currently preclude large-scale deployment beyond specialized aerospace applications, necessitating breakthroughs in low-cost, near-net-shape manufacturing processes.

### 3.2. Extreme Environments

**Figure 3:**
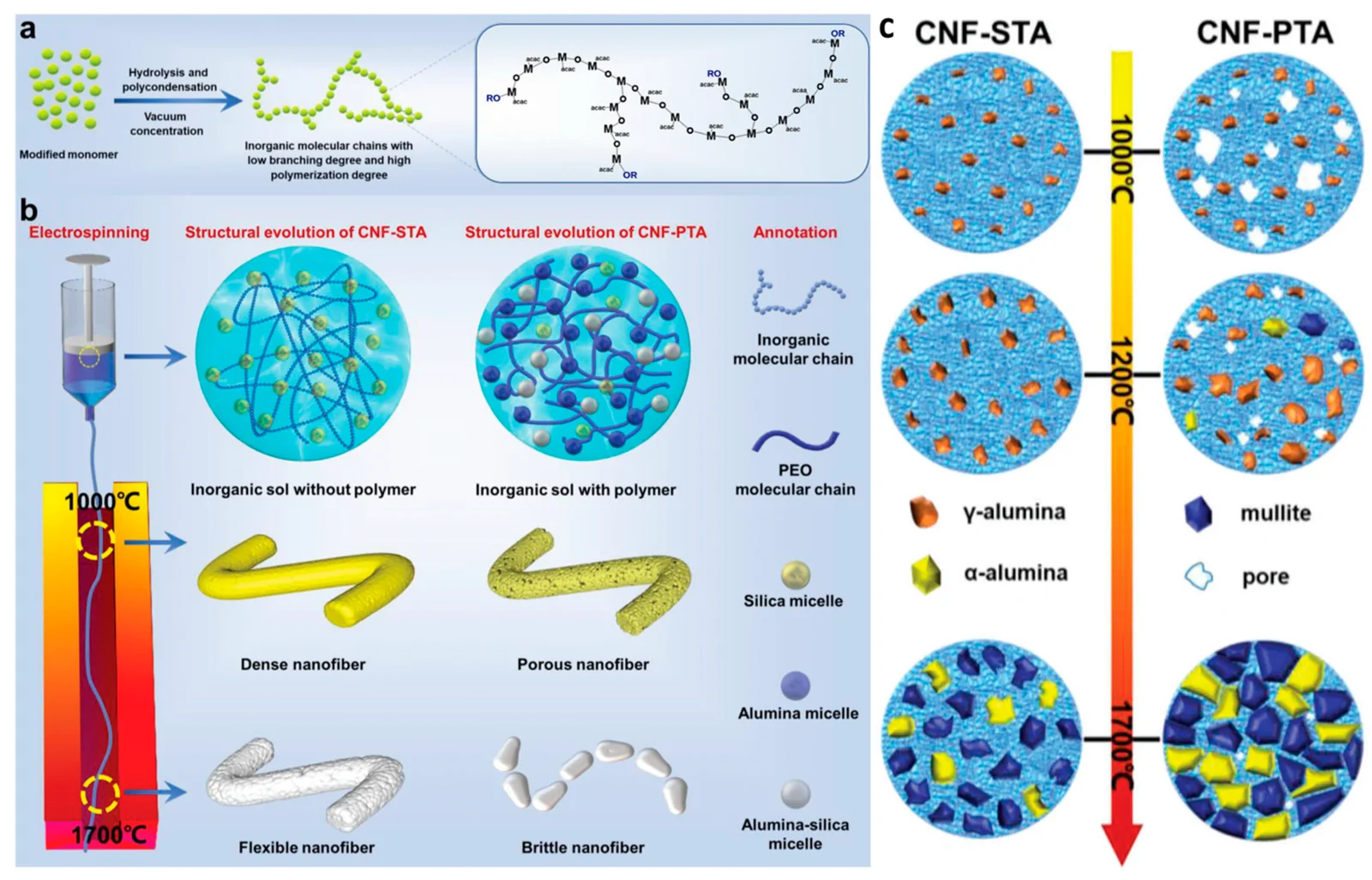
Synthesis of CNF-STA by the self-templated electrospinning method. (**a**) Evolution process and proposed molecular structure of IMC-LBHP. (**b**) Schematic illustration showing the difference between the traditional polymer-templated electrospinning method and our self-templated electrospinning method. (**c**) The difference in microstructures of CNF-STA and CNF-PTA calcined at typical temperatures (reprinted with permission from Ref. [49]).

Ceramic fibers and their derived nanofibrous architectures have emerged as a cornerstone for extreme-environment applications—including aerospace thermal protection, hypersonic skins, infrared stealth, and fire-resistant sensing—because they combine intrinsic high-temperature stability with tailorable microstructures that can be engineered for both thermal insulation and load bearing [18,40,49,50]. A central bottleneck above 1500 °C is detrimental grain growth and pore-defect accumulation during ceramization, which trigger embrittlement and structural collapse. Recent work addresses this challenge through molecular-chain-level precursor design and phase-transition modulation, which eliminate polymer templates, suppress localized pegging effects, and retard the γ-to-α Al_2_O_3_ transition. Consequently, tensile strengths near 1 GPa and macroscopic flexibility are retained even after exposure to 1700 °C (Figure 3) [49]. Parallel advances in nanograin–glass dual-phase configurations and template-free sol–gel electrospinning of TiO_2_ fibers relax the historic strength–deformability trade-off, unlocking approximately 1.06 GPa strength, approximately 8.4% strain, and even room-temperature plasticity in intrinsically brittle oxides at the single-fiber scale [30]. For conformal or articulating subsystems, programmable kirigami-inspired binary-network topologies convert rigid silica-zirconia ceramic aerogels into stretchable meta-aerogels. These structures achieve reversible tensile strains up to 85%, ultra-low thermal conductivity (approximately 33 mW m^−1^ K^−1^), and fatigue resistance across −196 to 1100 °C, extending ceramic insulation to movable aerospace skins [40]. Functionally, the same fiber platform enables extreme-condition sensing, where TiC–SiC membranes sustain 2000 °C and exhibit flame-driven piezoresistive response at approximately 1300 °C [18]; infrared stealth, where multiscale Al_2_O_3_–mullite/SiCNP aerogel–foil shields maintain a cold-face radiative temperature near 65 °C at 1500 °C [50]; and fire-safe radiative cooling, where all-inorganic Al_2_O_3_ membranes resist flame, UV aging, and combustion without polymer-driven degradation [43]. Collectively, these works reframe ceramic fibers not as brittle fillers but as architectured, load-bearing, thermal-function-integrable skins whose performance ceiling is determined by grain-boundary stability, interfacial defect chemistry, and topological design, rather than by bulk brittleness alone.

Extreme environments impose conflicting demands: ceramics must retain strength and flexibility while resisting grain growth, oxidation, and thermal shock—requirements that push single-phase materials to their limits and necessitate multiscale defect engineering. Although ceramic fibers exhibit remarkable retention of flexibility and strength at temperatures up to 1700 °C, their long-term cyclic stability under realistic hypersonic flight conditions—where thermal shock, aerodynamic shear, and oxidative erosion occur simultaneously—remains insufficiently characterized. Nevertheless, the vast majority of studies evaluate fiber performance under idealized laboratory heating profiles, neglecting the synergistic degradation caused by concurrent thermal cycling, mechanical vibration, and oxidizing atmosphere encountered in service. Most existing evaluations rely on short-term static tests, which fail to capture the progressive interfacial degradation between fiber matrices and functional coatings.

### 3.3. Electronic Textiles and Multifunctional Electronic Skin

**Figure 4:**
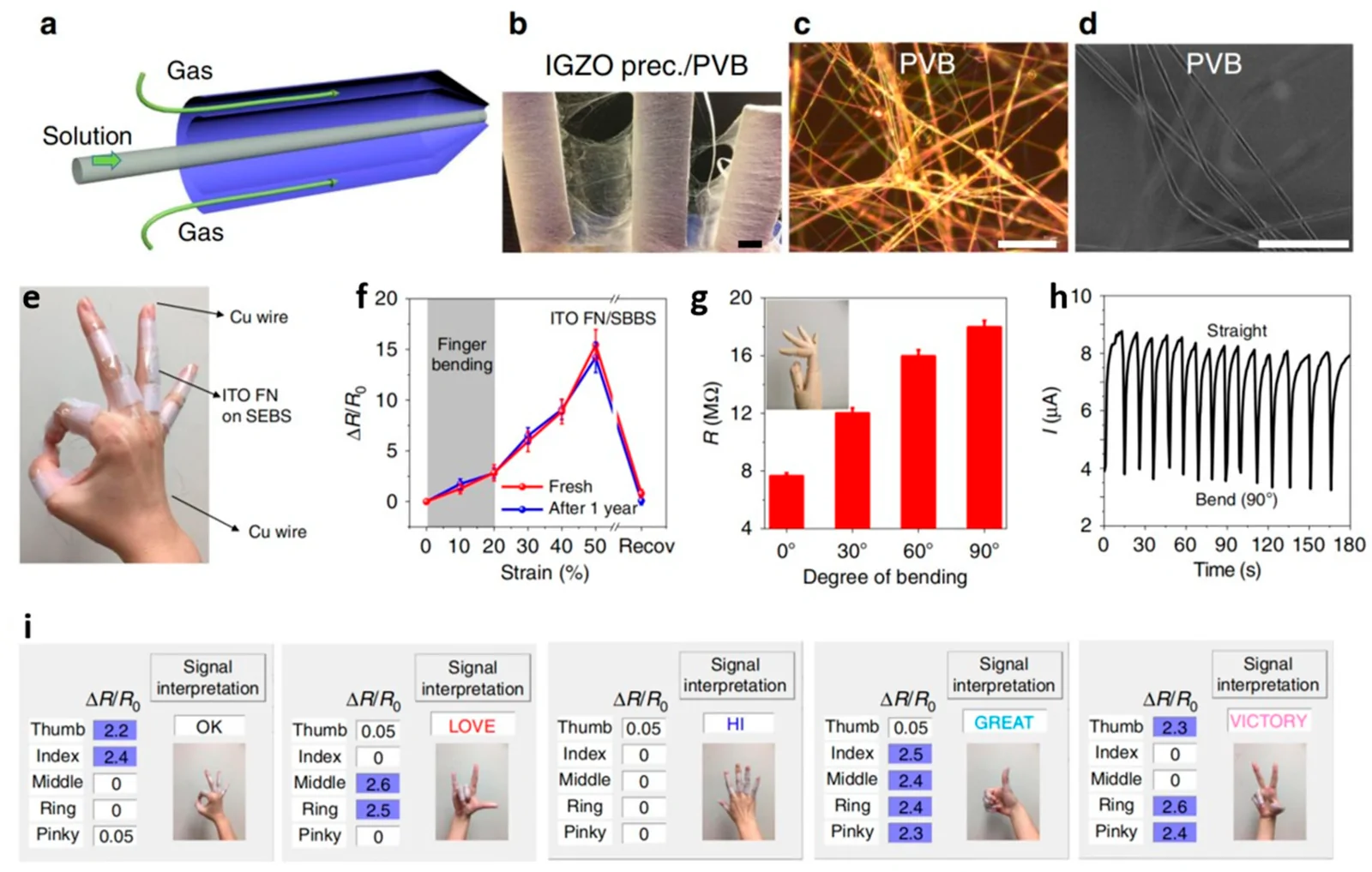
Synthesis of fibers and sensing under differentiating stimuli. (**a**) Schematic of the blow-spinning method. (**b**) Photograph of a metal salt/PVB fiber mat of IGZO fibers. Scale bar = 1 cm. (**c**) Optical image PVB fibers without metal salts. Scale bar = 50 μm. (**d**) SEM image of PVB fibers without metal salts. Scale bar = 10 μm. (**e**) Photograph of a hand fitted with ITO FN-based sensing devices. (**f**) Resistivity change of fresh and 1-year-storage ITO FN-based devices under different strains. (**g**) Electrical resistance of ITO FN-based devices under different degrees of finger bending. (**h**) Cycling test for wearable ITO FN-based devices bending from 0° to 90°. (**i**) Demonstration of an array of strain sensors placed on a hand to translate gestures (reprinted with permission from Ref. [65]).

Ceramic fibers have historically been excluded from truly flexible and wearable platforms, because bulk oxide crystals fracture at strains far below those imposed by daily bending or stretching. A central design pivot, therefore, is to reframe ceramic “brittleness” from an immutable atomic property into a structurally engineerable problem of mesoscale assembly and stress redistribution. In this vein, electrospun oxide crystal (TiO_2_, ZrO_2_ and SiO_2_) nanofibers can be rendered knottable and membrane-foldable by enforcing a brick-and-mortar motif, in which orderly assembled nanocrystals (the bricks) are bridged by twin or amorphous grain boundaries (the mortar). This architecture enables bending to accommodate local dislocation slip and compliant boundary elasticity rather than catastrophic crack propagation [11]. Complementary to purely ceramic routes, scalable blow-spun metal-oxide nanofiber networks (IGZO, CuO, ITO, Cu) exploit percolative charge transport within a three-dimensional fibrous mat to deliver mechanically tolerant building blocks. These networks sustain repeated bending (e.g., approximately 1000 cycles) and support room-temperature gas sensing, stretchable resistors, and monolithically integrated e-skin architectures capable of discriminating strain, pressure, temperature, humidity, and breath-related signals (Figure 4) [65]. For wearables that must also survive harsher duty cycles and added functionality, core–shell wet-spun ceramic fibers decouple robustness from brittleness. An aramid nanofiber (ANF)-based shell preserves continuity and toughness, while cold isostatic pressing compacts the conductive Al-doped ZnO ceramic nanoparticle core to raise contact-dependent sensitivity (e.g., gauge factor of approximately 2141) and broaden temperature readout. Additionally, this configuration enables fabric-level impedance matching for X-band electromagnetic attenuation, establishing a stealth-capable e-skin concept [68]. Collectively, these examples reframe ceramic fibers not as doomed-to-crack ribbons but as architectured, mesostructure-governed elements whose flexibility, sensing capability, and environmental resilience can be co-designed for next-generation electronic textiles.

Transforming intrinsically brittle ceramics into flexible, wearable platforms requires not only nanoscale architectural design but also a fundamental rethinking of how stress is redistributed across hierarchical interfaces—a challenge that current strategies address only partially. Ceramic fiber-based electronic skins exhibit exceptional sensitivity and high-temperature tolerance; however, their operational lifespan under physiological conditions remains a critical concern. Importantly, the reported sensitivity and durability are typically measured under pristine laboratory conditions; the effects of sweat corrosion, detergent washing, and repeated mechanical deformation on long-term signal fidelity remain largely unexplored. Challenges such as sweat-induced ion migration, protein fouling on ceramic surfaces, and delamination of core–shell interfaces following repeated laundering are seldom addressed. Moreover, integrating these brittle ceramic fibers into conventional textile weaving machinery without compromising their structural integrity necessitates a delicate equilibrium between fiber toughness and textile processability—a balance that current material designs have yet to achieve on an industrial scale.

### 3.4. Wave Absorption

Ceramic fibers have emerged as a central building block for wave-absorption applications, owing to their high-temperature stability, tunable chemistry, and architected porosity [54,55,64]. These attributes enable simultaneous electromagnetic wave (EMW) dissipation and acoustic-wave energy loss within a single material family. On the microwave/radar front, SiOC-based ceramic fibers engineered with Fe single-atom/Fe-N_x_ sites and nitrogen-doped carbon domains shift the dominant absorption band toward the low-frequency C-band while preserving oxidation resistance up to ≥500 °C. By coupling conduction loss, abundant interfacial and dipole polarization, and improved impedance matching—rather than relying on vulnerable magnetic nanoparticles—these fibers achieve ultra-thin-thickness performance with an effective reflection loss (RL) of approximately −59 dB [55]. On the acoustic side, three-dimensional ceramic nanofibrous/spongy architectures derive broadband absorption not solely from chemical composition but from hierarchically opened and closed cells, lamellar tortuous channels, and multi-interface reflection [54,64]. These features prolong wave propagation paths and enhance viscous and frictional dissipation. A reported example demonstrated lightweight spongy SiO_2_-Al_2_O_3_ composite ceramics that exhibit notable noise-reduction coefficients while retaining elasticity over a wide temperature range and maintaining invariant compression resilience from −196 to 1000 °C [64]. Collectively, these works reframe ceramic fiber “wave absorbers” as porous, architectured media in which fiber diameter, layered/lamellar anisotropy, cell-wall sealing, and engineered defect/heterointerface chemistry are co-optimized to efficiently convert wave energy into heat under extreme environments.

Wave absorption in ceramic fibers is inherently a multi-physics problem: electromagnetic, acoustic, and thermal waves interact with the same porous architecture, yet most studies optimize for only one wave type at a time, leaving the potential for truly integrated stealth unrealized. Moreover, most existing studies concentrate on single-frequency-band performance, whereas real-world military applications require broadband compatibility—specifically, radar–infrared integrated stealth. Compounding this issue, the impedance matching required for broadband absorption often conflicts with the dense, highly crystalline microstructure needed for high-temperature strength—a trade-off that has not been systematically mapped. As a result, achieving synergistic impedance matching across multiple frequency bands while simultaneously preserving thermal insulation and load-bearing capacity remains a complex multi-objective optimization challenge that impedes practical implementation.

### 3.5. Others

**Figure 5:**
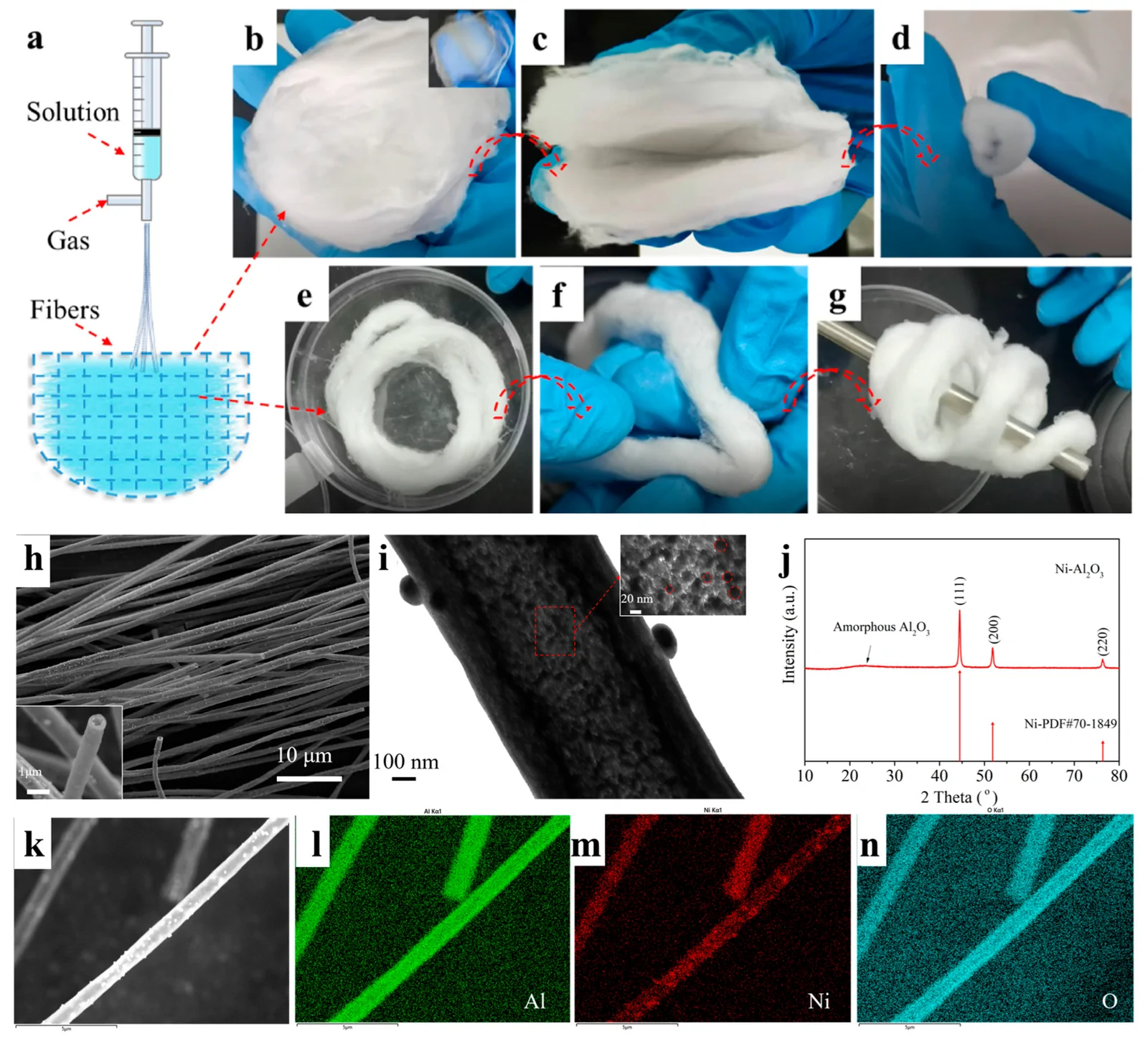
Ni/Al_2_O_3_ fibers for high-temperature catalysis. (**a**) Schematic diagram of the preparation of Al_2_O_3_ flexible fibers by blow spinning. (**b**) Photographs of laminated Al_2_O_3_ flexible fibers. (**c**) Folded membrane, (**d**) rolled membrane. (**e**) Photograph of round strip. (**f**) Folded round strip, (**g**) winded round strip of Al_2_O_3_ flexible fibers. (**h**–**n**) are morphological and phase composition of in situ Ni/Al_2_O_3_ fibers. (**h**) SEM pattern, (**i**) transmission electron microscopy (TEM) pattern, (**j**) X-ray diffraction (XRD) pattern, and (**k**–**n**) EDS graphical analysis (reprinted with permission from Refs. [66,67]).

Ceramic fibers are increasingly penetrating non-traditional territories where their high-temperature stability, tailored porosity, and scalable fibrous form factor create advantages that bulk ceramics or polymer textiles cannot match. A prominent frontier is fiber-based thermoelectrics. By exploiting ductile inorganic semiconductors and molten core drawing, superflexible Ag_2_Te_0.6_S_0.4_ fibers achieve tensile strains up to approximately 21.2% and retain stable output through roughly 1000 bending cycles. This enables true three-dimensional wearable fabrics that harvest body-ambient temperature differences, with normalized power densities approaching those of high-performance Bi_2_Te_3_ platforms [60]. In parallel, oxide ceramic textiles can be transformed from insulators into conductors at room temperature via domino-cascade Li-driven reduction. This unlocks lightweight, freestanding current-collector/electrode architectures (e.g., black TiO_2_ textiles) that combine flexibility with decent conductivity and improved cycling behavior in lithium batteries [56]. Beyond energy conversion and storage, ceramic fibers serve as high-temperature catalyst supports. Blow-spun Al_2_O_3_ fibers and their hollow Ni/Al_2_O_3_ analogs provide self-supporting, high-surface-area, chemically stable media for dry reforming of methane at ≥800 °C. Strong metal–support interaction and confined geometries mitigate sintering and coking, permitting regeneration for repeated use (Figure 5) [66,67]. Collectively, these examples demonstrate that the “other applications” of ceramic fibers are less about repurposing inert refractories and more about designing architectured, interface-rich fibrous networks that concurrently manage electrons, ions, heat, and reactive mass transport.

While ceramic fibers have expanded into thermoelectrics, batteries, and catalysis, these applications remain at an early proof-of-concept stage. Two critical gaps hinder further progress: first, the cyclic stability under realistic hydrothermal conditions (e.g., steam reforming) has yet to be rigorously evaluated; second, the scalability and mechanical integrity of fiber–electrode interfaces after thousands of charge/discharge cycles remain largely unexplored. These unresolved factors pose significant barriers to commercial viability, ultimately determining whether ceramic fibers can compete with established polymer- or carbon-based alternatives.

## 4. Challenges and Future Perspectives

Despite the remarkable progress achieved in the preparation, performance optimization, and application expansion of ceramic fibers, several critical challenges remain on the path toward their large-scale commercialization: Most high-performance ceramic nanofibers are currently produced using laboratory-scale electrospinning, which suffers from low throughput, time-consuming processes, and difficulties in ensuring the continuity of long-distance fibers. The development of scalable production techniques—such as high-speed centrifugal spinning, melt blowing, modified continuous melt spinning, and needle-free Kármán vortex street blow spinning—represents an inevitable trend for future advancement [29,35,38,63]. Although high-entropy engineering and phase-transformation control have partially mitigated high-temperature grain growth, the long-term creep resistance, thermal shock resistance, and environmental corrosion resistance of ceramic fibers under realistic service conditions (e.g., gas turbine engines or spacecraft surfaces subjected to moisture, oxygen, and high-speed airflow erosion) still require systematic evaluation [1,49]. Current ceramic fibers are predominantly designed for single or limited combinations of functions. Future efforts should leverage advanced materials genome engineering and AI-driven high-throughput screening to develop multifunctional smart ceramic fibers capable of simultaneously responding to multiple external stimuli (e.g., temperature, stress, and magnetic fields) and achieving programmable control over their functionalities [22,52]. This includes overcoming conventional physical constraints such as the coupling between high thermal conductivity and high electrical conductivity [53]. Many advanced ceramic fibers (e.g., carbides and high-entropy ceramics) involve high raw-material costs and energy-intensive sintering processes. Developing low-cost precursors, exploring low-temperature sintering mechanisms, and recycling waste ceramic fibers are key strategies for promoting green and sustainable development [36,38].

Recycling ceramic fibers presents significant technical hurdles. First, some ceramic fibers are multi-element composites (e.g., Bi_2_Te_3_, Ag_2_Te_0.6_S_0.4_, Ce_0.2_Sm_0.2_Gd_0.2_Nd_0.2_Y_0.2_)_2_Zr_2_O_7_, (Gd_1/2_Lu_1/2_)_2_(Ti_1/3_Zr_1/3_Hf_1/3_)_2_O_7_), making elemental separation and purification extremely difficult [23,60,61,76]. Second, their outstanding chemical stability—especially resistance to acids and alkalis—renders conventional chemical dissolution methods inefficient and environmentally burdensome due to large volumes of waste liquid [15,36]. Third, prolonged exposure to high temperatures during service induces grain growth, phase transformation, and microcrack propagation, leading to severe mechanical degradation that cannot be reversed by simple repair [49]. Promising recycling routes currently under investigation include: (i) mechanical comminution—crushing and milling waste fibers into nano- or submicron powders, which can then be used as fillers or reinforcements in new ceramic matrices or composites; (ii) re-sintering—mixing the milled powder with binders or fresh raw materials (e.g., sintering aids) followed by pressing and high-temperature firing to produce recycled ceramic parts; and (iii) selective leaching using mild acidic/alkaline solutions or molten salts, though process conditions must be carefully optimized to minimize environmental impact. These approaches remain at the laboratory stage and require further development for industrial scale-up.

SiC fibers (particularly third-generation near-stoichiometric, highly crystalline grades) are expected to attract the greatest attention and fastest growth due to their superior high-temperature oxidation resistance, creep resistance, and neutron irradiation stability [36,48]. Key applications include hot-section components of aerospace engines, nuclear reactor cladding, and first-wall structures in fusion reactors. High-performance alumina fibers (e.g., α-Al_2_O_3_) and zirconia fibers (e.g., Y_2_O_3_-stabilized ZrO_2_) offer excellent thermal insulation and chemical inertness at relatively moderate cost [15,77]. They will continue to see strong demand in industrial furnaces, automotive exhaust treatment, solid oxide fuel cells, and other high-temperature environments. Other non-oxide ceramic fibers, including boron carbide (B_4_C) [36], silicon nitride (Si_3_N_4_) [78], boron nitride (BN) [79], and borides (e.g., ZrB_2_) [20], although smaller in total volume, possess irreplaceable advantages under extreme conditions (ultra-high temperature, severe corrosion, neutron shielding). They will maintain steady development in niche but critical sectors such as defense, aerospace, and specialty chemicals.

## 5. Conclusions

Ceramic fibers bridge the gap between brittle bulk ceramics and textile-processable reinforcements primarily through drastic dimensional reduction: the markedly smaller characteristic dimension lowers the probability of critical flaw inclusion (a volume effect), thereby mitigating the flaw sensitivity inherent in bulk ceramics and endowing the fibers with sufficient flexibility for weaving and composite integration. Each spinning method offers a distinct kinetic pathway to realize this principle: electrospinning traps nanoscale fibers via rapid solvent evaporation; melt spinning exploits polymer-to-ceramic conversion kinetics; blow spinning enables hierarchical 3D assembly; and wet spinning provides a platform for seamless textile integration. The key scientific advance lies in decoupling mutually exclusive properties through molecular-level precursor engineering, phase-transition modulation, high-entropy stabilization, and bio-inspired architectures. These strategies collectively yield unprecedented combinations of mechanical strength, thermal stability, and multifunctionality—enabling applications from thermal superinsulation above 1500 °C to extreme-environment sensing at 2000 °C, infrared stealth, electromagnetic absorption, thermoelectric harvesting, and wearable electronics. However, significant mechanistic challenges remain: understanding long-term creep and thermal shock requires probing defect evolution under coupled thermomechanical–chemical fields; integrating multiple functions demands programmable interfaces rather than simple component mixing; and industrial scalability hinges on controlling precursor chemistry at scale. Future progress will rely on predictive design—using multiscale computation to link processing parameters directly to targeted microstructures and performance. Ceramic fibers are positioned to serve as a cornerstone material platform for next-generation aerospace, energy, and electronic technologies, uniquely combining thermal resilience, mechanical compliance, and functional integrability.

## Figures and Tables

**Table 1:** Electrospinning technology.

Materials	Diameter (μm)	Density (mg·cm^−3^)	Testing Temperature (°C)	λ (mW m^−1^ K^−1^)	Other Properties	Applications	References
Al_2_O_3_-SiO_2_	0.2–0.4	30	1600	50.58	N/A	Thermal protection	[2]
(Gd_1/2_Lu_1/2_)_2_ (Ti_1/3_Zr_1/3_Hf_1/3_)_2_O_7_	0.25	4.35	−196 to 1500	81.21 at 1000 °C	N/A		[23]
Si-C-N	1.0	N/A	2300	80 at 1200 °C	Absorption bandwidth (36 GHz)		[44]
Aluminosilicate	0.1–0.3	N/A	1400	103.55	N/A		[45]
La_2_Y_0.4_TiZr_2_O_9.6_	0.65	50.2	1300	21.96	N/A	Thermal superinsulation	[46]
Hypocrystalline zircon	0.7	15–55	1300	104	N/A		[47]
Mullite, HfO_2_, ZrO_2_, TiO_2_	2	5–20	1127	106.7	N/A		[39]
SiC@SiO_2_	0.5	27	1300	91.4	N/A		[48]
TiC-SiC	0.6	15	2000	N/A	N/A	Extreme environments	[18]
TiO_2_	0.35–0.45	N/A	N/A	N/A	Strength (1.06 GPa)		[30]
Al_2_O_3_	0.4	10	1700	N/A	N/A		[43,49]
	0.15–0.7	N/A	N/A	N/A	Cool power (75 W/m^2^)		
Silica-zirconia	379.3	36	1100	33.01	N/A		[40]
SiC@SiO_2_-Al_2_O_3_	0.2	10	1500	30.6	N/A		[50]
SiO_2_	0.3	N/A	N/A	N/A	Strength (1.41 GPa)	Thermal management	[8,21,51,52]
	0.49	7	1100	26	Heat flux (110.43 W cm^−2^)		
	0.35–1.2	300	1500	34	Electromagnetic shielding (−26.6 dB at 3.5 mm)		
	0.5–5	4	1300	26.1	N/A		
C/SiCON	0.9	100	1000	19.8	N/A		[53]
(La_0.2_Y_0.2_Nd_0.2_Gd_0.2_Sr_0.2)_CrO_3_	0.46	N/A	1300	140–420	Cool power (75 W/m^2^)		[22]
TiO_2_, ZrO_2_ and SiO_2_	0.35	N/A	1000	N/A	Bending rigidity (22 mN)	Electronic textiles	[11]
SiO_2_/GO	0.5	N/A	550	9.3	Noise reduction (0.56 in 63–6300 Hz)	Wave absorption	[54]
SiOC-Fe-CN	0.25	N/A	1000	N/A	Wave loss (−58.0 dB at 5.93 GHz)		[55]
TiO_2_, SnO_2_, BaTiO_3_	0.322	1.78	1000	N/A	N/A	Li battery	[56]

N/A: not available; λ: thermal conductivity.

**Table 2:** Melt-, blow- and wet-spinning technologies.

Technologies	Materials	Diameter (μm)	Density (mg·cm^−3^)	Testing Temperature (°C)	λ (mW m^−1^ K^−1^)	Other Properties	Applications	References
Melt spinning	Ag_2_Te_0.6_S_0.4_	300	N/A	827	0.0004 at 20 K	Thermoelectric (mV and 559 nW)	Thermoelectric performance	[60]
	Bi_2_Te_3_	4	N/A	700	3.18 at 2.6 K	Figure-of-merit value (1.4)		[61]
	Tb^3+^-doped glass–ceramic	400	N/A	20 to 400	N/A	Sensitivity (224 nGy/s); stability (over 240 cycles)	X-ray imaging and flexible detection	[62]
	Graphene fiber (GF) and TiC coating	25	N/A	2200	745,000 for single fiber	Mass ablation rate (0.3 mg s^−1^)	Thermal protection	[29]
Blow spinning	ZrO_2_ (TiO_2_, YSZ, BaTiO_3_)	0.18	8–40	1300	27	N/A	Thermal insulation	[3,17]
		0.45	5	−196 to 1300	28	N/A		
	SiO_2_, Al_2_O_3_ ZrO_2_, TiO_2_	0.07	N/A	1300	N/A	Sorption weight gain (20,000%)		[63]
	Al_2_O_3_/SiO_2_ core–shell	0.28	8	−196 to 1300	7	N/A		[6]
	SiO_2_-Al_2_O_3_ composite	2.7	10	−196 to 1000	34	Sound absorption properties (NRC of 0.77)	Acoustic absorption	[64]
	In-Ga-Zn oxide	5.0	N/A	300	N/A	Bending radius (1 mm); sensitivity (33.6% ppm^−1^)	Versatile wearable electronics	[65]
	Mullite	0.36	2.18 to 20	−196 to 1500	28 to 88	N/A	Extreme environment	[19]
	Ni/Al_2_O_3_	1.0	N/A	−196 to 1200	57 at 400 °C	N/A	Thermal catalysis	[66,67]
Wet spinning	GDOOH	0.001	N/A	N/A	N/A	Elongation (86%)	Optical, electrical fields	[16]
	Al-doped ZnO	200	N/A	N/A	N/A	Reflection loss (−39.1 dB)	Electronic skin	[68]
	ZrB_2_-SiC	0.5	4.27 to 4.7	0 to 1200	N/A	Ablation rates (0.34 mg/s)	Thermal protection	[20]

N/A: not available; λ: thermal conductivity.

## Data Availability

The original contributions presented in this study are included in the article. Further inquiries can be directed to the corresponding authors.
